# GCN-Based LSTM Autoencoder with Self-Attention for Bearing Fault Diagnosis

**DOI:** 10.3390/s24154855

**Published:** 2024-07-26

**Authors:** Daehee Lee, Hyunseung Choo, Jongpil Jeong

**Affiliations:** 1 Department of Electrical and Computer Engineering, Sungkyunkwan University, 2066 Seobu-ro, Jangan-gu, Suwon-si 16419, Gyeonggi-do, Republic of Korea; hahalee98@kakao.com; 2 Department of Smart Factory Convergence, Sungkyunkwan University, 2066 Seobu-ro, Jangan-gu, Suwon-si 16419, Gyeonggi-do, Republic of Korea

**Keywords:** bearing fault diagnosis, fault simulator, graph convolution network (GCN), self-attention, long short-term memory (LSTM) autoencoder

## Abstract

The manufacturing industry has been operating within a constantly evolving technological environment, underscoring the importance of maintaining the efficiency and reliability of manufacturing processes. Motor-related failures, especially bearing defects, are common and serious issues in manufacturing processes. Bearings provide accurate and smooth movements and play essential roles in mechanical equipment with shafts. Given their importance, bearing failure diagnosis has been extensively studied. However, the imbalance in failure data and the complexity of time series data make diagnosis challenging. Conventional AI models (convolutional neural networks (CNNs), long short-term memory (LSTM), support vector machine (SVM), and extreme gradient boosting (XGBoost)) face limitations in diagnosing such failures. To address this problem, this paper proposes a bearing failure diagnosis model using a graph convolution network (GCN)-based LSTM autoencoder with self-attention. The model was trained on data extracted from the Case Western Reserve University (CWRU) dataset and a fault simulator testbed. The proposed model achieved 97.3% accuracy on the CWRU dataset and 99.9% accuracy on the fault simulator dataset.

## 1. Introduction

At present, the manufacturing industry operates within a constantly changing and developing technology environment, and it is very important to ensure that any manufacturing processes in this environment are efficient and reliable. Rapidly detecting and responding to various failures that may occur in the manufacturing process can directly contribute to improving productivity and reducing costs. Motor-related failures occupy a large proportion of manufacturing processes, and bearing faults [1] represent one of the most common and fatal problems.

Bearings are mechanical elements that provide accurate and smooth movements, and they play an important role in mechanical equipment with shafts. Because of this importance, many studies have focused on diagnosing bearing failure [2,3,4]. However, due to the imbalance of abnormal data and the complexity of time series data, it remains difficult to diagnose bearing failures in manufacturing. This has led to the use of existing AI models such as the convolutional neural network (CNN) [5], long short-term memory (LSTM) [6], support vector machine (SVM) [7], and extreme gradient boosting (XGBoost) [8] to limit fault diagnosis.

The methods that are currently used to maintain machines in the manufacturing industry [9] can be largely divided into post-maintenance, preventive maintenance, and predictive maintenance. Post-maintenance is a method of taking measures after the machine breaks down, and it has enormous costs in terms of productivity and finances. Preventive maintenance is a method of performing maintenance at a set period, although it has the disadvantage of incurring regular maintenance costs. Pre-maintenance predicts and measures machine failure to prevent its occurrence, primarily by monitoring equipment conditions using various sensors such as vibration, temperature, and camera sensors [10], and it detects abnormal signs in advance, allowing for maintenance or repair. Pre-maintenance can minimize production interruptions or cost increases stemming from unexpected failures.

However, several problems must be solved before prediction and maintenance can be effectively implemented. The first is the imbalance between normal and abnormal data. In actual manufacturing sites, there is generally much less abnormal data than normal data, which makes it difficult to learn the model. The current paper solved this problem by generating various bearing fault data using a directly constructed fault simulator testbed. The second is the accuracy problem that artificial intelligence models struggle with. To diagnose bearing faults with high accuracy, the graph convolutional network (GCN)-based self-attention LSTM autoencoder model was proposed. An experiment using this model on the Case Western Reserve University (CWRU) open dataset [11] showed 97.3% accuracy. Moreover, the accuracy of the proposed model was 99.9% when tested on the dataset extracted from the directly constructed fault simulator testbed. These results verified the reliability and excellence of the proposed model.

The major contributions of this paper can be summarized as follows:

- A fault simulator testbed was constructed to extract and utilize various bearing data.
- A bearing fault diagnosis model that can be used in various situations was developed by using the CWRU dataset, which is an open dataset, along with directly extracted data.
- The proposed model achieves 97.3% accuracy using the CWRU dataset, which is an open dataset, while it achieves 99.9% accuracy using the data that have been extracted through the fault simulator testbed.

The rest of this paper is organized as follows: Section 1 contains an introduction to detail the research purpose and research methodology. Section 2 reviews related work and explains the time series data analysis techniques and various deep learning models used therein. Section 3 elucidates the bearing failure diagnosis method proposed in this paper. Section 4 presents the experimental environment and experimental results. Finally, Section 5 synthesizes the experimental results and discusses future research plans.

## 2. Related Work

### 2.1. Types of Failures in Rotating Machines

Rotating machines are used as core pieces of equipment in various industrial fields, and their efficient operation is one of the important factors that is directly connected to the productivity of a product. Rotating machines play an essential role in converting energy into mechanical power, and they are used in various industrial environments such as turbines, generators, fans [12], and motors [13]. Failure of a rotating machine is a factor that hinders the growth of the industry, as it causes economic losses to the enterprise due to problems such as serious productivity degradation, increased maintenance costs, and equipment shutdown [14].

**Figure 1:**
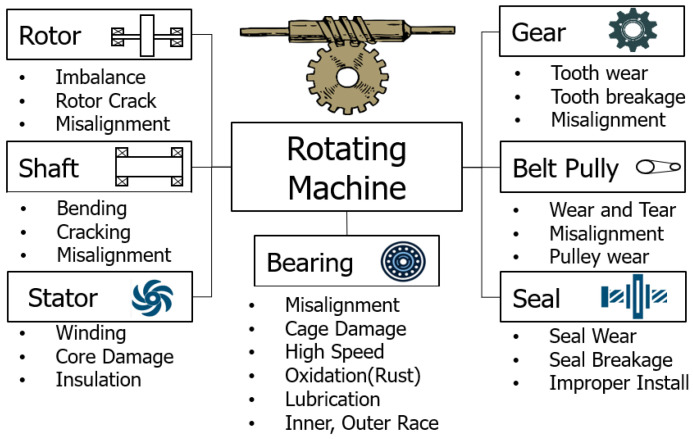
Types of failures in rotating machines.

There has recently been a significant increase in researchers [15] examining the diagnosis of failures in rotating machines using artificial intelligence. Such research focuses on the development of artificial intelligence models that can systematically analyze various types of failures that may occur in rotating machines such as rotors [16], gears [17], shafts [18], bearings [19], stators [20], belt pulleys [21], seals [22], etc. This makes it possible to quickly diagnose and predict failures. These models enable the early detection of failures, precise analysis of their causes, and efficient response strategies—all of which contribute to prolonging the machine’s lifespan and minimizing maintenance costs. Figure 1 shows the various types of failures that can occur in rotating machines; these types of failures are highly diverse, and their characteristics vary depending on the unique role and operating environment of each component. For example, faults in bearings are mainly caused by inner races, outer races [23], and damage to carriages [24], and these failures lead to poor overall performance of the machine. Meanwhile, imbalances or cracks in the rotor increase the vibration and noise of the system, thereby lowering the efficiency and safety of the machine. This paper aims to precisely analyze the types of faults in bearings, particularly in rotating machines, and develop an effective fault diagnosis model.

### 2.2. Bearing Fault Diagnosis

Bearings are some of the key components of a rotating machine, and the failure of a bearing can significantly affect the performance of the entire machine. It is essential to diagnose bearing faults to detect these failures early, and it is also crucial to accordingly perform proper maintenance to improve the reliability and lifespan of the machine. There are various causes of bearing faults, such as misalignment [25], inner races, outer races, high-speed damages [26], damage to carriages, foreign body insertion, lubrication, and oxidation [27]. Early detection of the causes of these various faults is a very important part of ensuring continuous operation of the machine and maintenance of productivity. Various methods can be used to diagnose bearing faults, and typical methods include vibration analysis [28], sound analysis [29], and temperature monitoring [30].

Vibration analysis is the most commonly used method to monitor the state of a bearing. Vibration analysis involves evaluating the state of a bearing by analysis conducted in the frequency domain, and it captures changes occurring in vibration signals according to major causes of failure. The primarily used techniques include fast Fourier transform (FFT) [31], short-time Fourier transform (STFT) [32], and wavelet transform (WT) [33]. These methods can be used to diagnose failure by analyzing frequency components, and they can be used to accurately determine the normal and abnormal states of bearings while considering data continuity and periodicity.

Sound analysis is a method in which failures are diagnosed by analyzing the acoustic signals generated from a bearing. This is achieved utilizing noise analysis and ultrasonic analysis using an acoustic sensor, and abnormal noise such as abrasion or cracks generated inside the bearing can be detected through this process. In particular, acoustic analysis is more effective when it is used together with vibration analysis.

### 2.3. Deep Learning Techniques for Bearing Fault Diagnosis

Bearing fault diagnosis—which uses deep learning, a technique in the field of machine learning—is a method of monitoring the condition of a bearing and identifying a failure by analyzing large amounts of data obtained from a sensor. This method plays an important role in detecting and preventing problems before a failure occurs. Throughout the data collection process, sensors collect various physical signals that are generated by the bearing, such as vibration, temperature, and sound. The data collected in this way are used as basic data for bearing fault diagnosis and play an important role in the next step (the data pre-processing process).

Next, the data that have been collected from the sensor are converted into forms that are suitable for analysis through a pre-processing process before being used for further analysis. The process includes noise removal [34], normalization [35], and feature extraction in the time-frequency domain. The characteristics of the data that have undergone such pre-processing are reinforced, and unnecessary information is removed, thus increasing the accuracy of model learning. After that, the deep learning model is designed and trained. Complex data patterns are learned using various deep learning models, such as CNN, LSTM, and autoencoder. These models learn the features that are necessary to distinguish between normal and fault states in a large amount of data and to diagnose the state of the bearing based on this. The learned model can be applied to new sensor data to detect anomalies and predict the type of faults in the bearing.

Recent studies have utilized various deep learning techniques to improve the performance of bearing fault diagnosis. Z. Dong et al. [36] proposed a bearing fault diagnosis method by combining empirical wavelet transform (EWT) and one-dimensional improved self-attention-enhanced convolutional neural network (1D-ISACNN). The method was experimented on three different bearing test benches and achieved 100% accuracy in all cases. This study combined feature extraction in the frequency domain with a self-attention mechanism, which greatly improved the accuracy of bearing failure diagnosis. W. Li et al. [37] combined bidirectional long short-term memory (BiLSTM) and CNN to perform the tool residual life prediction. The model can learn bidirectional information from time-ordered data, and can accurately predict the remaining life by monitoring the status of the tool in real time. This study shows that combining the strengths of LSTM and CNN can effectively learn patterns in complex time series data.

In this paper, we propose a GCN-based LSTM autoencoder model utilizing self-attention. Unlike previous studies, the proposed model utilizes STFT instead of the wavelet transform, uses graph convolutional network (GCN) to extract spatial and structural features of the data, and combines the LSTM autoencoder with a self-attention mechanism to learn important time series patterns. This approach is advantageous for effectively detecting and diagnosing complex bearing fault patterns. The following deep learning models are utilized for bearing fault diagnosis.

#### 2.3.1. LSTM Autoencoder

**Figure 2:**
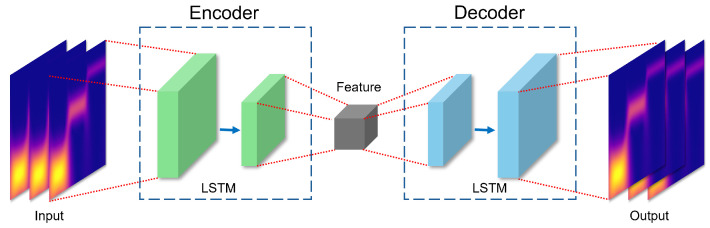
LSTM autoencoder model.

The LSTM autoencoder [38] is a neural network model that is designed to effectively encode and decode the features of time series data or sequential data. This model combines the time-dependent capture capability of LSTM with the data compression and reconstruction capabilities of the autoencoder, as shown in Figure 2. The LSTM autoencoder consists of an encoder and a decoder, which converts the input data into latent representations, and the decoder restores this latent representation to the original data. This allows the model to learn the important characteristics of the input data and minimize reconstruction errors.

This model is used for tasks such as anomaly detection, data compression, and sequence regeneration. In particular, the LSTM autoencoder has excellent performance in various applications, as it can effectively extract and restore important features of time series data. For example, in the anomaly detection task, the LSTM autoencoder can detect abnormal signs based on the difference between reconstruction errors between normal and abnormal data. In the data compression task, the data can be efficiently compressed while maintaining important information on the input data, and in the sequence regeneration task, the sequence of the original data can be accurately restored. These advantages have allowed the LSTM autoencoder to be effectively utilized in various fields, including financial data analysis, health monitoring, and predictive maintenance. Its ability to learn complex patterns of time series data and extract important features has also allowed this model to perform well in applications that require high degrees of both reliability and accuracy.

#### 2.3.2. Attention Mechanism

The attention mechanism technique [39] is an important technology used for processing sequence data in deep learning; it was first used in natural language processing (NLP) and has since come to be widely used in various fields. This mechanism helps the model learn important information better by weighting specific parts of the input sequence. There are forms such as self-attention [40], global attention, local attention, etc. In self-attention, all elements within the input sequence interact to capture important patterns, which are implemented as scaled dot-product attention and extended to multi-head attention. Global attention considers the entire sequence, while local attention only considers a few sections to increase computational efficiency.

The attention mechanism is effectively used in the Transformer model [41]. The Transformer captures important information about the input sequence through self-attention, and it consists of an encoder and decoder structure. This structure extracts information from various perspectives and generates the final output. The advantages of the attention mechanism are that it focuses on the important parts when processing long sequence data, thereby increasing computational efficiency, which ultimately allows it to be easily integrated into various model structures, and to effectively learn important patterns of the input sequence.

In conclusion, the attention mechanism provides excellent performance for sequence data processing in deep learning, and it is used in various fields such as natural language processing, speech recognition, and time series data analysis. The use of this mechanism improves the performance of the model and enables more effective learning of important information.

#### 2.3.3. GCN

A graph is a data structure that is composed of nodes and edges, where the nodes represent entities and the edges represent relationships between nodes. Graphs are useful for representing various types of data, and they are particularly effective in modeling correlations between data points. The K-nearest neighbor (K-NN) [42] algorithm is a method that can be used to find and connect K closest neighbors for each data point, and ultimately construct a graph based on the similarity between data points. This makes it possible to effectively model the spatial and structural relationship of data. Graph composition using K-NN is particularly advantageous for visualizing and analyzing correlations between unstructured data or time series data.

GCN [43] is a model that specializes in learning the node characteristics of graph data, and it updates the characteristics of each node by combining them with the characteristics of that node’s neighboring nodes. GCN utilizes the structural information of the graph to enhance the representation of nodes, through which it learns the patterns and interactions in the graph. The main idea of GCN is to define the convolution operation in the structure of the graph. This convolution operation is similar to the convolution operation that is used in image processing, but it reflects the structural characteristics of the graph. The formula for the GCN layer is as follows:

(1) $$H^{\left(l+1\right)}=\sigma \left({\tilde{D}}^{-1/2}\tilde{A}{\tilde{D}}^{-1/2}H^{l}W^{l}\right)$$

Here, $H^{l}$ is the node characteristic matrix of layer *l*. $\tilde{A}=A+I$ is the adjacent matrix of the graph, which includes connections to the node itself. $\tilde{D}$ is the diagonal matrix of $\tilde{A}$. $W^{l}$ is the learnable weight matrix of layer *l*. σ is an activation function (e.g., rectified linear unit (ReLU)).

The GCN updates the node characteristics in each layer by combining them with the characteristics of its neighboring nodes. In the first layer, the initial node characteristics are accepted as input, while in the subsequent layer, the node characteristics are gradually updated using the output of the previous layer as input. Through this process, the global structure information of the graph is gradually learned.

Since GCN operates on graph data, if the input data are not in the form of a graph, it is necessary to first convert them into a graph; the K-NN algorithm can be used for this process. For each data point, K-NN is used to find K neighbors, and each data point is set as a node of the graph, while the connection of neighbors found through K-NN is set as an edge of the graph.

The graph generation process using K-NN is as follows: First, the distance between each data point is calculated. In general, the Euclidean distance or cosine similarity is used. Then, for each data point, the nearest K neighbors are found. Finally, a graph is generated by setting each data point as a node and neighbors found through K-NN as edges. The graph and node characteristic matrices generated in this way are input to the GCN model, while the characteristics of the node are updated and learned through the GCN layer.

GCN can be used in a variety of applications. For example, it can be used to analyze the relationship between users in social networks, to predict the properties of compounds by analyzing molecular structures, or model the interaction between users and items in recommendation systems. These properties of GCN ultimately make it possible to perform pattern recognition and prediction in graph data in a more efficient manner.

### 2.4. STFT

**Figure 3:**
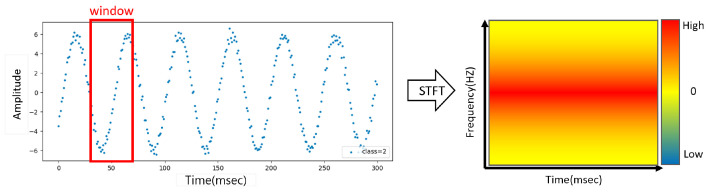
STFT model.

STFT is a technology that has been developed to overcome the limitations of FFT, and it involves dividing a signal into short time intervals and then applying the Fourier transform to each interval. This method can obtain the frequency components of the signal in each time interval, and by arranging them along the time axis, it is possible to observe the frequency change over time. STFT divides the signal into several short intervals, which generally have overlapping parts to maintain the continuity of the signal. Figure 3 shows the STFT transformation. Since it can select and analyze time intervals of various lengths, it has the advantage that it can be applied in a flexible manner according to the characteristics of the signal to be analyzed. STFT is very useful when the characteristics of the signal change over time, as is the case in real-time signal processing or music and audio signal analysis. However, depending on how the length of the interval is set, there may be a trade-off between the time resolution and the frequency resolution.

## 3. GCN-Based LSTM Autoencoder with Self-Attention Model

**Figure 4:**
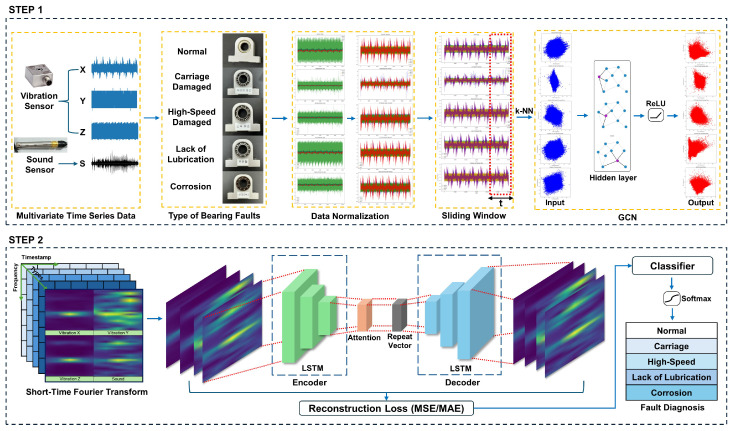
GCN-based LSTM autoencoder with self-attention model.

This chapter proposes a GCN-based LSTM autoencoder with self-attention model to bear fault diagnosis using multivariate time series data. The proposed model consists of two steps, as shown in Figure 4. This process can successfully diagnose the failure of bearing data.

### 3.1. Step 1

**Figure 5:**
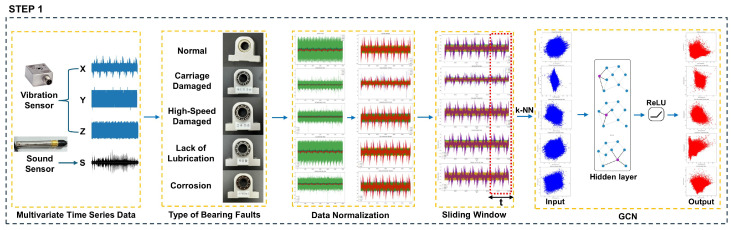
Step 1.

**Figure 6:**
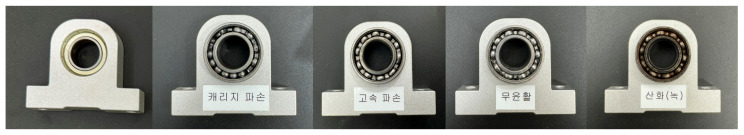
Types of bearing faults.

Figure 5 shows step 1, which visually represents the data pre-processing step. In this step, data including various fault states and steady states are loaded, and standardization and normalization are performed as well. Based on various types of bearing equipment, as shown in Figure 6, the data extracted from the three-channel vibration sensor and the sound sensor having the X, Y, and Z axes are normalized to make it suitable for analysis.

Normalized data are divided into certain sizes using the sliding window technique. The sliding window technique divides time series data into fixed-length windows and uses each window as one sample, while the fixed window size and slide are adjusted to divide the data, thus allowing the model to learn data patterns in various time intervals.

The K-NN algorithm is then used to find the k nearest neighbors for each data point, and a graph is generated based on the similarity between the data points. This process makes it possible to effectively model the spatial and structural relationship of the data. The generated graph is learned through the GCN layer.

GCN is a neural network structure that is optimized for processing graph data, and it extracts new characteristics by considering the characteristics of each node and its neighboring nodes together. This makes it possible to learn complex patterns and structural information of data and to effectively extract important features from the frequency domain of time series data. The main advantage of GCN is that it can be learned while maintaining the relationship between each data point by utilizing the graph structure, which makes a substantial contribution to increasing the accuracy of fault diagnosis. The frequency domain data obtained through this process are then transferred to step 2.

### 3.2. Step 2

**Figure 7:**
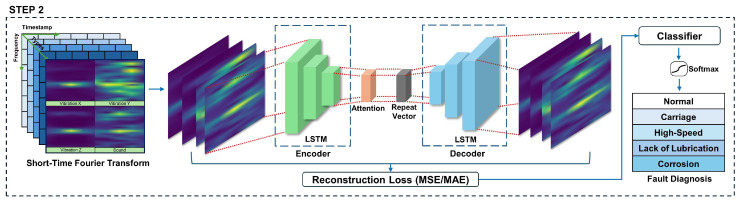
Step 2.

Step 2 is shown in Figure 7, which represents the model experiment stage. It begins with the process of converting the features extracted through GCN, the last step of step 1, into STFT for analysis in the frequency domain. In this step, the data are analyzed by converting the temporal characteristics of the time series data into a frequency domain. STFT can analyze the frequency change pattern over time by dividing the time series data into short time intervals and then performing the Fourier transform for each section. Using this process, the spatial and structural characteristics obtained through GCN can be expressed in terms of changes in time and frequency. GCNs are used to extract spatial and structural characteristics of time series data. The network learns the features of each node through its relationship with its neighbors, which is advantageous for identifying multidimensional patterns in the data. GCNs are powerful tools that reflect the inertia and interactions of data based on their graph structure, which is especially useful for complex network structures.

The LSTM autoencoder with a self-attention layer simultaneously learns the long-term and short-term dependencies of the time series data. In this model, self-attention plays a role in assigning weights to important parts of the encoder’s output sequence [44]. The LSTM encoder creates a hidden state of each sequence while processing time series data, and it applies self-attention to assign weights to important parts. The self-attention mechanism calculates the importance of all time steps that are different from itself for each time step in the sequence, and it then weights them based on these calculations to emphasize important information in order of relative importance. These weights are determined through learning, and they are gradually adjusted while the model learns. The self-attention mechanism applies a probability function to estimate a score from the bottleneck vector representation. The estimated score is multiplied by the bottleneck vector representation to obtain a context vector. The context vector is then input to the decoder and reconstructed into the original encoder input data dimension. The computation method uses M. Luong et al.’s global attention concatenation multiplicative technique [45], which allows the model to emphasize important information and learn important features of time series data more effectively. The self-attention layer is defined in the following way:

(2) $$e_{i}=\tanh\left(W\cdot x_{i}+b\right)$$

(3) $$a_{i}=\frac{\exp(e_{i})}{\sum_{j}\exp\left(e_{j}\right)}$$

(4) $$o_{i}=\sum_{j}a_{j}\cdot x_{j}$$

In the above equation, $x_{i}$ represents a vector corresponding to a time step *i* of an input sequence, while *W* and *b* are learnable weights and biases. $e_{i}$ is the score representing the importance of each time step, and $a_{i}$ is the weight that is normalized through the softmax function. Finally, $o_{i}$ is the sum of weighted input vectors, which represents an output vector emphasizing important information. By defining the self-attention layer in this way, an abnormal state may be detected through the process of assigning weights to important portions of the output sequence of the encoder and transferring them to the decoder to reconstruct the original data.

The important features weighted by self-attention are converted into repeat vectors and passed to the decoder. The decoder is responsible for reconstructing the original data based on the features extracted from the encoder. The decoder uses the last state of the encoder as the initial state to generate a sequence, which is used to calculate the reconstruction error and detect anomalies. The main role of the decoder is to restore the original shape of the input data based on the information learned from the encoder.

The output layer of the model is responsible for the classification of failure types. The output layer consists of a fully connected (dense) layer, which uses CrossEntropyLoss as the loss function to train the model. For multi-class classification, the LeakyReLU and Softmax activation functions are used to predict the probability for each type. This allows the model to distinguish between normal and abnormal data and accurately classify different defect types. While many previous studies mainly used the ReLU activation function, the proposed model uses the LeakyReLU activation function to perform multi-class classification. To enable the model to learn complex patterns, we set LeakyReLU in the hidden layer to add nonlinearity. LeakyReLU solves the dead ReLU problem by maintaining a small gradient when the output of a particular node is negative. This makes learning more stable. Softmax is set on the output layer of a multi-class classification to convert the predicted values for each class into a probability distribution by making the output value equal to 1. The Softmax activation function calculates the log-likelihood of each class, converts it to an exponential function, and normalizes the sum to 1 to generate the final probability distribution. This process allows the model to learn the features of the input data and accurately predict the probability for each class. Finally, the CrossEntropy Loss function is used to train the model and optimize the classification performance.

Through the feature learning and self-attention mechanism of GCN, the important parts of the time series data can be highlighted, which greatly improves the accuracy of fault diagnosis. The proposed model learns the patterns of normal and abnormal data and detects anomalies in the test data through reconstruction errors to provide an early diagnosis of bearing failure in multivariate time series data. This model has the advantage of achieving significantly improved fault diagnosis accuracy by highlighting the important parts of time series data through graph-based spatial characteristic learning and attention mechanisms. This helps effectively extract and learn important information, particularly from complex time series data.

The proposed model also evaluates the performance by comparing the reconstruction loss with mean squared error (MSE) and mean absolute error (MAE). MSE is a value that is averaged by squaring the difference between predicted and actual values, and it gives a greater penalty to the large error. MAE is the average of the absolute difference between the predicted and actual values, and it deals with all errors equally. By comparing the two indicators, the reconstruction capability and fault detection performance of the model can be evaluated in a more detailed manner. Through this process, the reliability and superiority of the proposed model are verified.

## 4. Experiment and Results

### 4.1. Experimental Setup and Datasets

Table 1 presents the experimental environment. The experiment described in this paper was conducted on a Windows workstation with an Intel Core i7-8700K CPU and an NVIDIA GeForce RTX 3080 GPU. Python version 3.7.7, TensorFlow 2.11.0, and CUDA version 11.1 were used.

#### 4.1.1. CWRU Dataset

Data provided by CWRU were used to verify the effectiveness of the proposed model. The CWRU dataset is a dataset that is widely used for bearing fault diagnosis and consists of a steady state, internal race failure, external race failure, and ball failure. The data were measured at locations such as the drive end, fan end, and base, and the depth of the defect varies between 0.007 inches, 0.014 inches, and 0.021 inches. The CWRU dataset consists of 12 kHz and 48 kHz drive-end-bearing datasets and 12 kHz fan-end-bearing data; this paper used drive-end data that were collected at 48 kHz. Specifically, data that were collected when the load of the motor was 2 horsepower (HP) was used. At this time, the shaft rotation speed of the motor was 1750 revolutions per minute (RPM).

**Figure 8:**
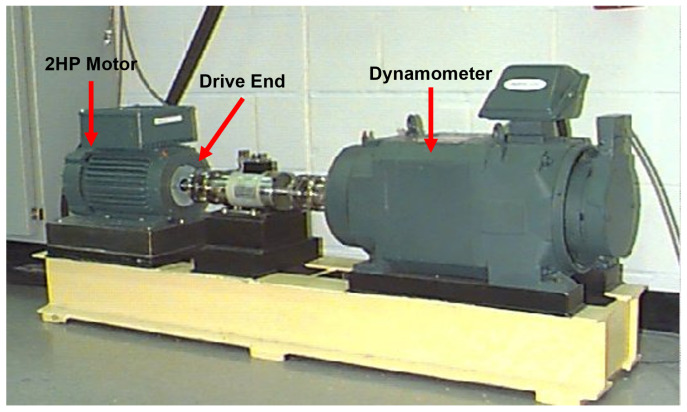
CWRU testbed.

The data used consist of 10 types: normal state, three types of ball defects (0.007 inches, 0.014 inches, and 0.021 inches), three types of internal lace failures (0.007 inches, 0.014 inches, and 0.021 inches), and three types of external lace failures (0.007 inches, 0.014 inches, and 0.021 inches) in the 6 o’clock position. This dataset contains bearing fault data under various conditions, which makes it suitable for evaluating the generalization performance of fault diagnosis models. The CWRU testbed is depicted in Figure 8.

The data were processed, made into a comma-separated variable (CSV) file format, and used. For failure identification prediction, nine feature values were calculated: maximum value, minimum value, average value, standard deviation, root mean square (RMS), skewness, kurtosis, crest factor, and shape factor. Each feature value was calculated in a time interval of 2048 points. These feature values were calculated for each segment and ultimately stored in a CSV file format.

Table 2 lists information on the dataset used in this experiment. Classifications for 10 types were diagnosed, and the train, validation, and test datasets were used at a 6:2:2 ratio for each type, respectively, while the data type numbers after STFT conversion were 340, 110, and 110, respectively. The hyperparameters used in this experiment are presented in Table 3.

#### 4.1.2. Fault Simulator Dataset

**Figure 9:**
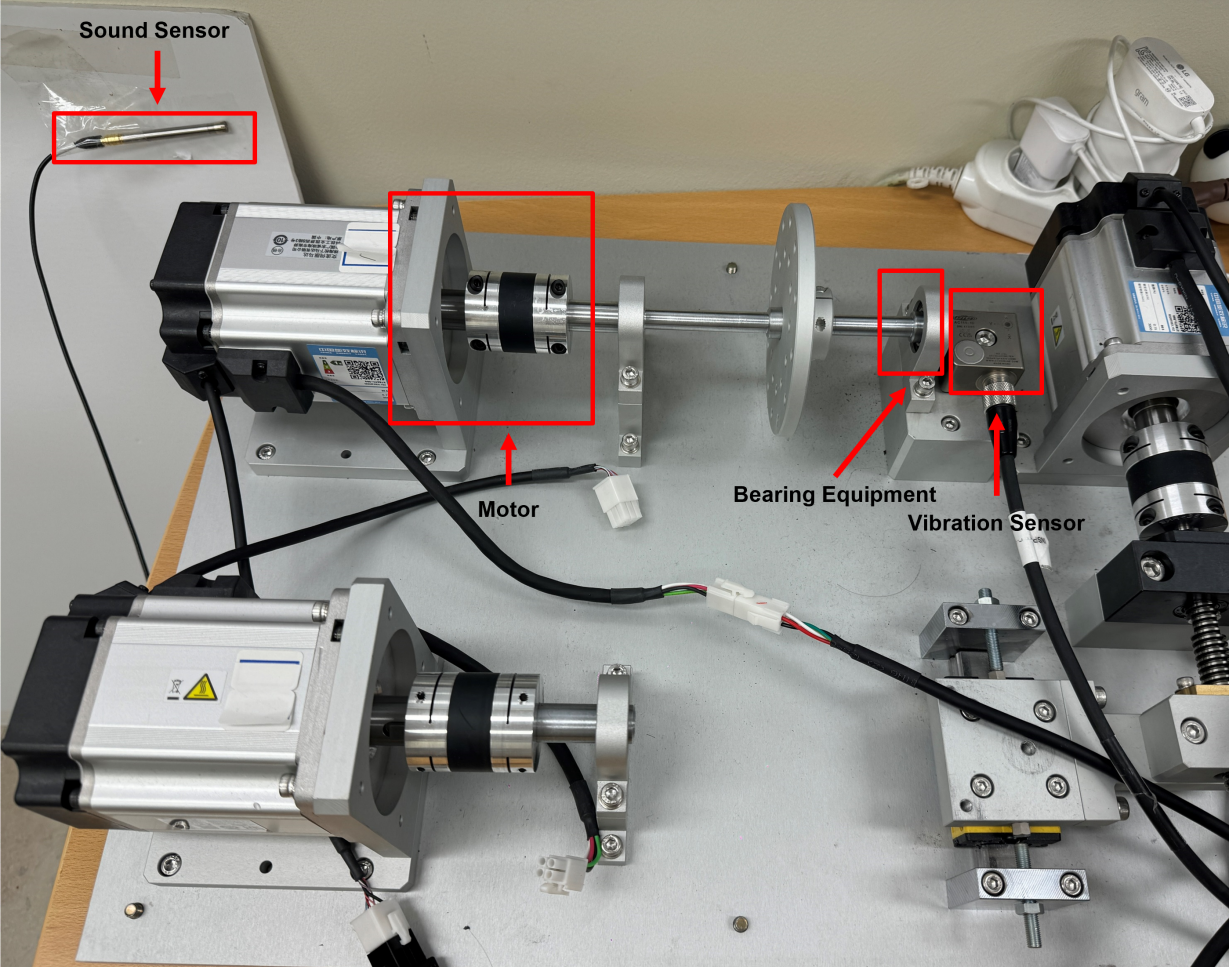
Fault simulator testbed.

**Figure 10:**
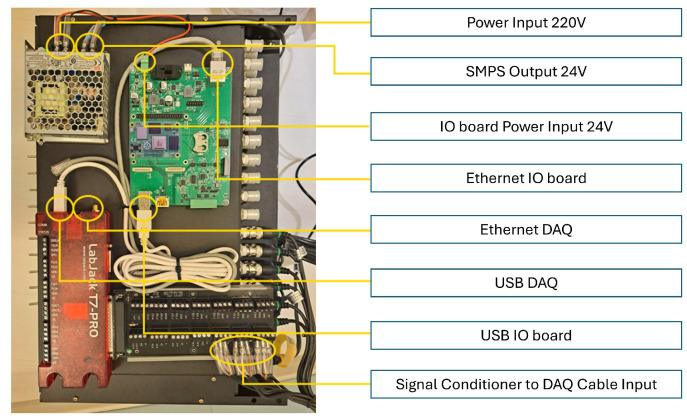
MQTT protocol and DAQ system.

In this paper, the fault simulator test bed shown in Figure 9 was constructed to collect data for bearing fault diagnosis. This test bed was designed to simulate various fault conditions and normal conditions. The message queuing telemetry transport (MQTT) protocol and data acquisition (DAQ) system shown in Figure 10 were used for data collection.

**Figure 11:**
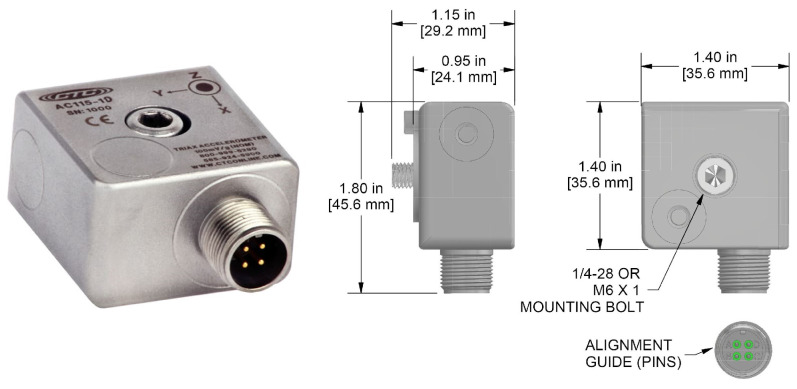
Vibration sensor.

**Figure 12:**
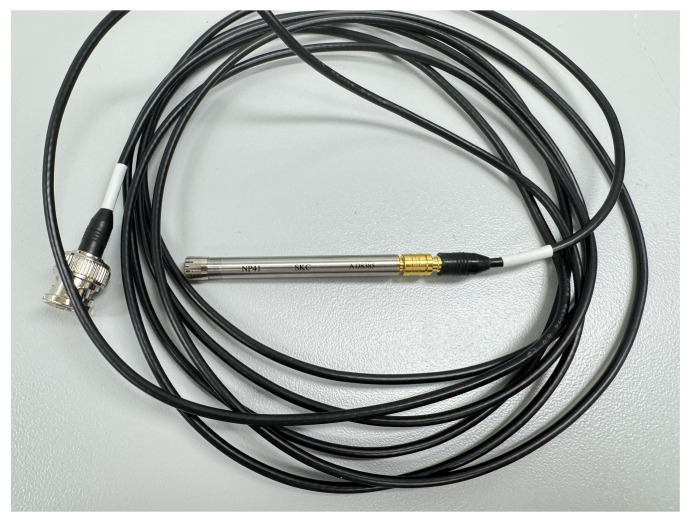
Sound sensor.

The test bed consists of equipment that can simulate various failure conditions that might affect bearings. This test bed can reproduce both the normal state and the failure state by replacing bearings, and it can collect vibration and sound data that is generated in each state in real time. To construct the test bed, three-axis vibration data were collected using a vibration sensor called AC115 from the Connection Technology Center company in Figure 11. Sound data were also collected using MP41, a microphone sensor from the Korean SI company, as shown in Figure 12. This sensor is capable of measuring high-precision sound, and it is used in the present work to measure the sound that is generated during bearing operation.

The data collected through the vibration and sound sensors were transmitted to the central server in real time through the MQTT protocol; MQTT is a lightweight message protocol that can efficiently transmit data in a limited environment such as a sensor network. Data transmission was performed by posting data from each sensor to the MQTT broker and receiving it from a central server. The DAQ system played a role in converting analog signals into digital data and transmitting it to a computer. Vibration and sound data were collected at a high speed through the DAQ system, while the data were collected at a sampling rate of 5000 Hz for 10 min, and the vibration and sound sensor values were stored in the CSV form with labels such as motorx, motory, motorz, sound, and time.

In this paper, bearing data were collected for 10 min by setting the rotational speed of the motor to 1500 RPM. To accurately grasp the defective sound of the bearing equipment, a sound sensor was attached to the bearing equipment and used at a position of 20 cm; when extracting the data, the data were extracted in an environment without noise other than the sound generated by the bearing. The collected bearing data were divided into five types: a normal state, high-speed damage, carriage damage, lack of lubricant, and oxidized state. Normal state data indicate a situation in which the bearing operates smoothly without failure, while high-speed damage was conducted with a bearing that has failed by rotating at high speed. Carriage damage was a state in which internal components of the bearing were damaged, and lack of lubricant was simulated as a state in which abrasion and heat were generated due to the absence of lubricant in the bearing. Finally, oxidized and corroded bearings were used in the oxidized state. Table 4 presents detailed information on the dataset used in this experiment. Each data type was used for training, validation, and testing in a respective ratio of 6:2:2, and the data numbers after STFT conversion are as follows:

The collected data were classified by each state, and they were treated using a pre-processing process. Sensor values (motorx, motory, motorz, and sound) were standardized and normalized, which had the effect of adjusting the scale of the data and improving the accuracy of the analysis. Time series data were divided into fixed-length windows through sliding windows, each window was used as one sample, and the data were divided by adjusting the window size and the slide. This process allowed the model to learn data patterns in various time intervals. The data were divided into window-extracted features in the frequency domain through STFT conversion, while the frequency domain data obtained through this process was used as the input of the model.

Table 5 presents the hyperparameters of the GCN-based LSTM autoencoder with a self-attention model. Various hyperparameters were tested using grid search, and the following settings showed the best performance. The hyperparameter values of the model are as follows.

### 4.2. Evaluation Metrics and Visualization

#### 4.2.1. Evaluation Factors

This paper used various indicators to evaluate the performance of the model in terms of bearing fault diagnosis. In particular, the prediction performance of the model was confirmed in an intuitive manner using the confusion matrix. The confusion matrix consists of the following four elements: true positive (TP) means the case where the actual value is true and is correctly predicted as true by the model. True negative (TN) means the case where the actual value is false and is correctly predicted as false by the model. False positive (FP) means the case where the actual value is false but the model incorrectly predicts it as true. Finally, false negative (FN) is the case where the actual value is false but the model incorrectly predicts it as true. Based on these evaluation factors, various important evaluation indicators may be calculated. First, accuracy represents the percentage of samples correctly predicted by the model. It is calculated as the ratio of samples accurately predicted by the model to the total number of samples, and it is expressed using the following formula:

(5) $$\mathrm{Accuracy}=\frac{TP+TN}{TP+FN+FP+TN}$$

Recall refers to the percentage of true positive values that the model correctly identifies as such. Reproducibility refers to how well the model finds the case where it is actually positive. High reproducibility means that the model does not miss the case where it is true; it is very important to increase reproducibility for use in certain applications. Recall is calculated using the following equation:

(6) $$\mathrm{Recall}=\frac{TP}{TP+FN}$$

Precision refers to the ratio of predicted values that are correctly identified as true. High precision means that there are many cases where the model is actually true among the predictions that have been made. Reproducibility and precision exist in a trade-off relationship, and increasing one can lower the other. Precision indicates how accurate the model’s prediction is, and it is expressed by the following formula:

(7) $$\mathrm{Precision}=\frac{TP}{TP+FP}$$

The F1 score is a harmonized average of precision and reproducibility, and if the data label is unbalanced, the performance of the model can be evaluated more accurately. This helps reliably evaluate the predictive performance of the model on an unbalanced dataset. The F1 score is calculated using the following formula:

(8) $$F1\text{-}\mathrm{Score}=2\times \frac{\mathrm{Precision}\times \mathrm{Recall}}{\mathrm{Precision}+\mathrm{Recall}}$$

#### 4.2.2. Reconstruction Loss

In this paper, we also evaluate the reconstruction loss of the autoencoder using MSE and MAE. MSE is the squared average of the difference between the predicted and actual values. Since the error is squared, it is more sensitive to large errors. The formula for MSE is as follows, where n is the total number of samples, $y_{i}$ is the actual value, and $\hat{y_{i}}$ is the predicted value.

(9) $$MSE=\frac{1}{n}\sum_{i=1}^{n}{\left(y_{i}-\hat{y_{i}}\right)}^{2}$$

MAE is the average of the absolute values of the differences between the predicted and actual values. It gives equal weight to all errors, and it is calculated using the following formula, where n is the total number of samples, $y_{i}$ is the actual value, and $\hat{y_{i}}$ is the predicted value.

(10) $$MAE=\frac{1}{n}\sum_{i=1}^{n}\left|y_{i}-\hat{y_{i}}\right|$$

These metrics are used to evaluate how well a model’s predictions match the true value, and each metric can help analyze a model’s performance in different ways according to its particular characteristics. MSE is more sensitive to large errors, which is useful when it is important to minimize large errors, while MAE treats all errors equally, which is useful when the aim is to evaluate overall prediction accuracy.

#### 4.2.3. PCA

**Figure 13:**
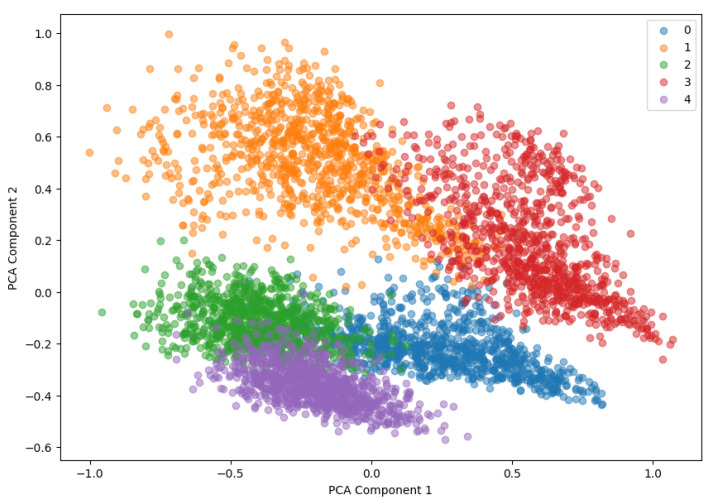
The 2D PCA.

High-dimensional data were visualized in two and three dimensions using principal component analysis (PCA). Through this, the embedding vector of the data learned by the model can be visually checked and the data distribution for each class can be identified. PCA is a technique that is used to visualize the structure of data by converting high-dimensional data into a low-dimensional space. The two-dimensional PCA visualization is shown in Figure 13, and the data distribution for each class can be visually checked in a two-dimensional space. This allows one to see how well the model separates each class.

**Figure 14:**
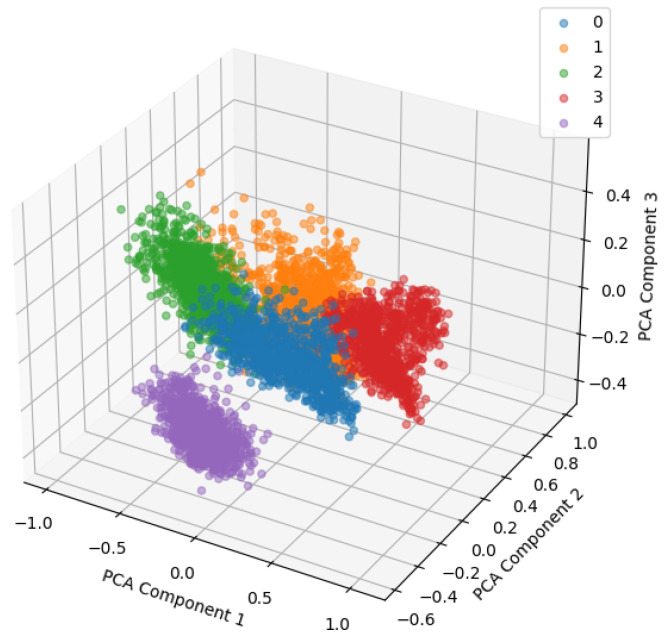
The 3D PCA.

The 3D PCA visualization is shown in Figure 14, and one can visually check the data distribution for each class in a 3D space. This makes it easier to grasp the structure of the data and the clustering performance of the model.

Through this PCA visualization, it is possible to analyze the embedding vector of the data learned by the model, and the performance of the model may be evaluated from various angles. Through this, the performance of the bearing fault diagnosis model may be comprehensively evaluated and improved.

### 4.3. Results

In this paper, performance comparisons with various comparative models were conducted to evaluate the performance of the proposed GCN-based LSTM autoencoder with a self-attention model. Accuracy and the F1 score were used as evaluation indicators, and the performance of each model was compared. As comparison models, LSTM, ConvLSTM, and LSTM autoencoder were used.

**Figure 15:**
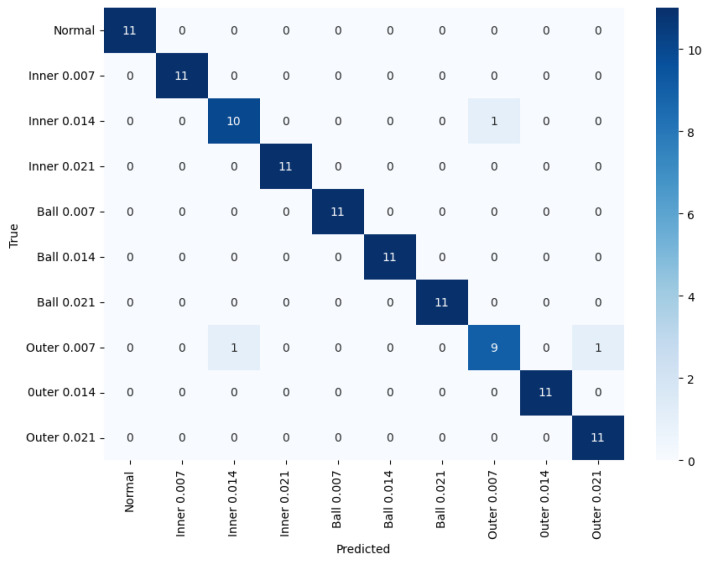
Confusion matrix for the CWRU dataset.

The performance of the proposed GCN-based LSTM autoencoder with a self-attention model was compared with other models using CWRU data. Table 6 presents the performance of the proposed model and other models. Figure 15 shows the confusion matrix for the suggested model.

**Figure 16:**
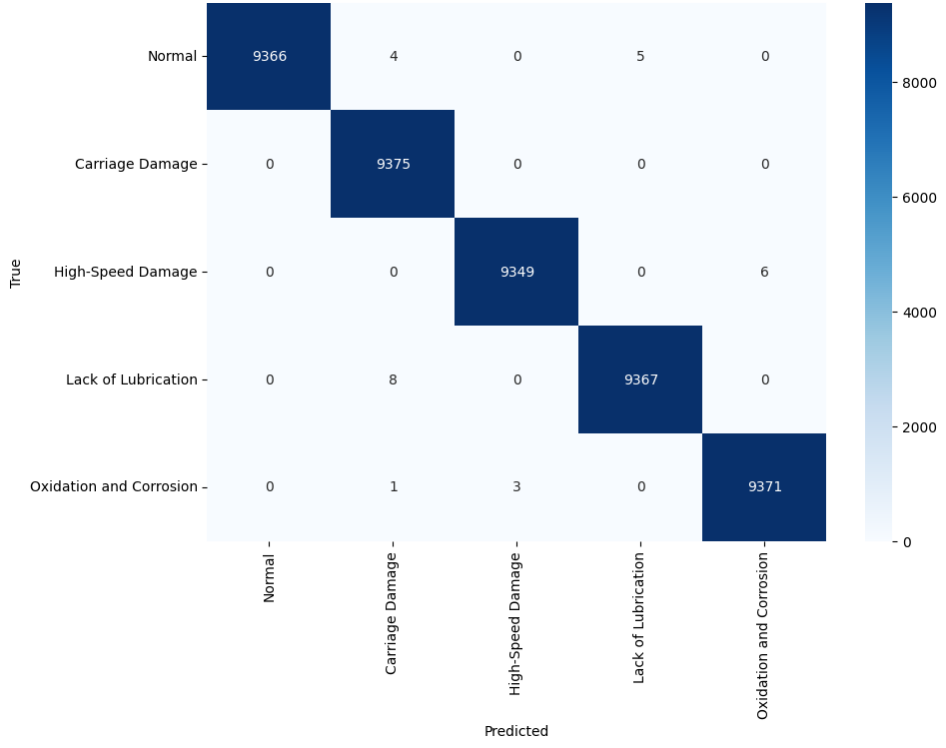
Confusion matrix for the fault simulator dataset.

The performances of the proposed model and several models were compared using data that was extracted from the directly constructed fault simulator testbed. Table 7 presents the performance of each model. The proposed model also showed high performance in directly extracted data, particularly when GCN and STFT were used together. Figure 16 shows the confusion matrix for the proposed model. As can be seen from the confusion matrix, the proposed model accurately classified most fault states and normal states.

**Figure 17:**
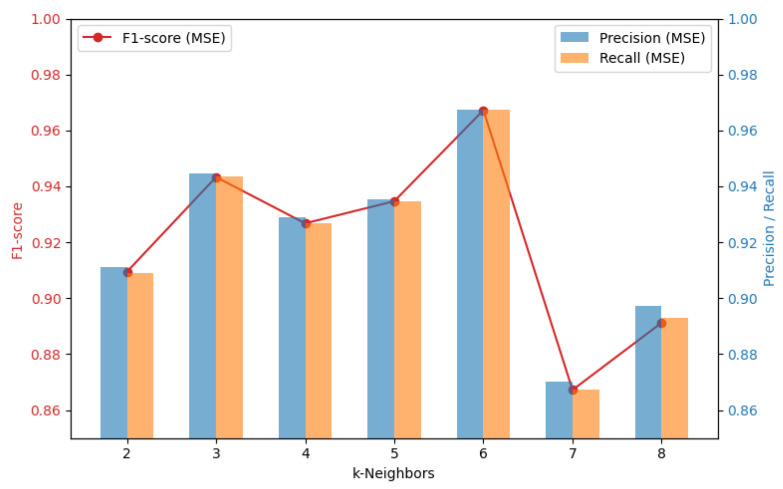
MSE values for different K-neighbors.

**Figure 18:**
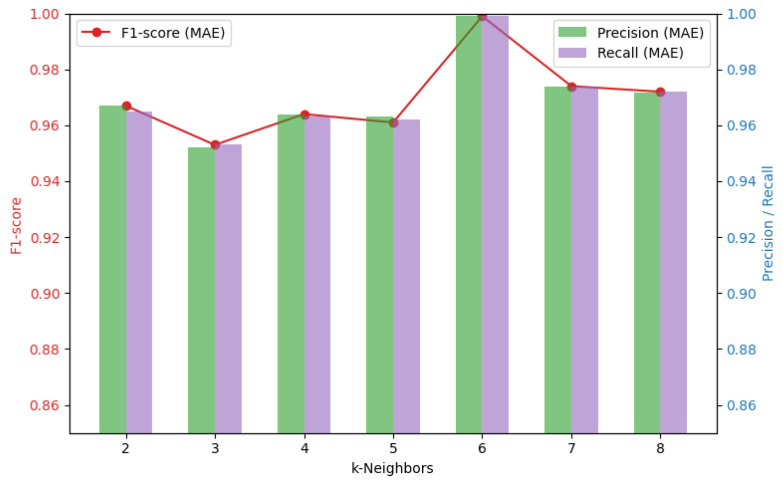
MAE values for different K-neighbors.

The reconstruction loss of the autoencoder was evaluated using MSE and MAE according to the K-neighbor value. Both indicators play an important role in evaluating the reconstruction performance of the model, and the results are shown in Figure 17 and Figure 18. Comparing MSE and MAE for each K-neighbor value, the minimum F1 score splits the resulting value of 0.864 and performs better with MAE than with MSE. It performs best when the K-neighbor is 6.

**Figure 19:**
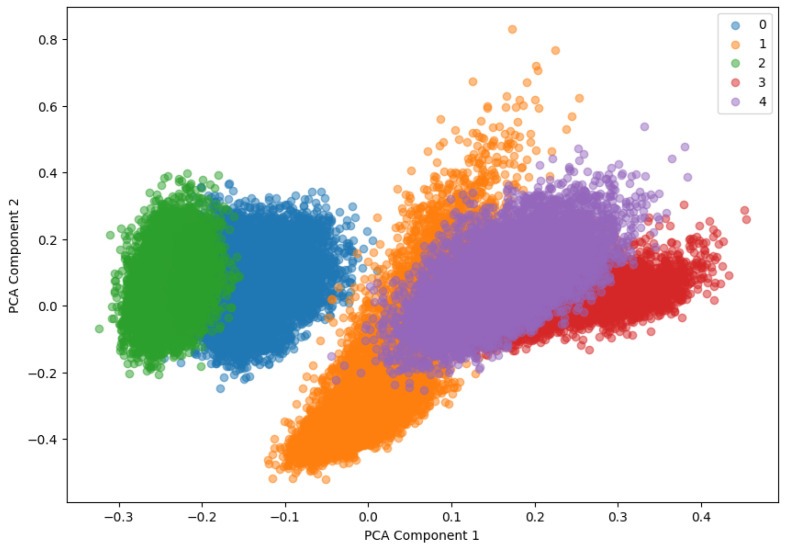
PCA of the fault simulator test dataset (2D).

Moreover, in this paper, high-dimensional data were visualized in both two and three dimensions using PCA. The two-dimensional PCA visualization is shown in Figure 19, and the data distribution for each class can be visually confirmed in a two-dimensional space. This made it possible to confirm how well the model separates each class.

**Figure 20:**
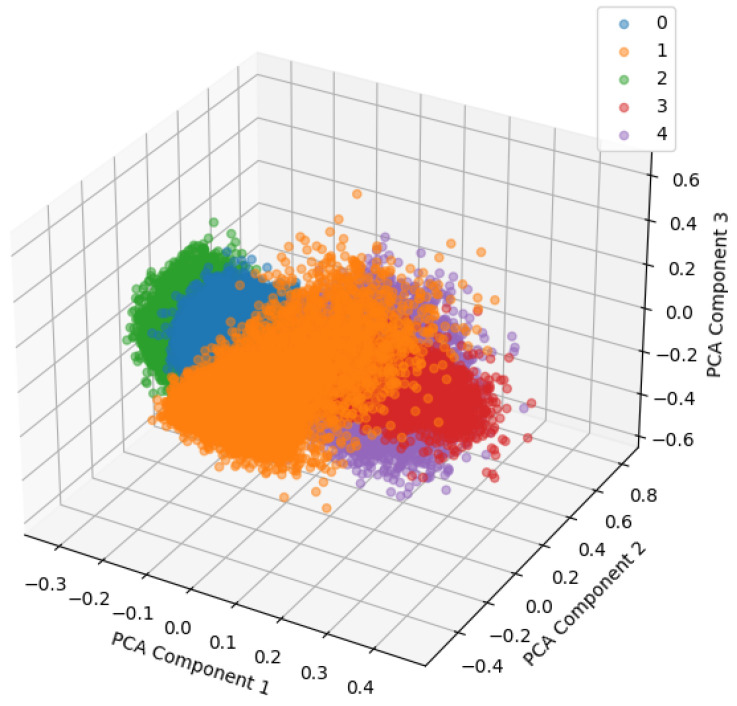
PCA of fault simulator test dataset (3D).

As shown in Figure 20, the 3D PCA visualization made it possible to visually check the data distribution for each class in a 3D space. This allowed for a clearer understanding of the data structure and the clustering performance of the model.

As a result of the experiment, the proposed GCN-based LSTM autoencoder with the self-attention model showed superior performance compared to other comparison models on the CWRU dataset, which is an open dataset, and the directly constructed fault simulator data. In particular, the proposed model exhibited high acuity and F1 score through reconstruction loss (MSE, MAE) evaluation and confusion matrix analysis. Moreover, by visually analyzing the embedding vector of the data learned by the model through 2D PCA visualization and 3D PCA visualization, the clustering performance of the model could be evaluated. This confirmed how effectively the proposed model separated the data of each class.

The proposed model recorded 99.9% accuracy in early diagnosis of bearing fault in multivariate time series data, thus outperforming the other models. In particular, compared to the accuracy of 97.3% achieved on the CWRU dataset, it has shown the potential to increase the applicability in real industrial sites by maintaining high performance in fault simulator data that is collected in real industrial environments.

These results may contribute to improving the reliability and lifespan of the machine. The proposed model greatly improves the accuracy and reliability of bearing fault diagnosis, and these results are expected to play an important role in establishing an early failure diagnosis system in industrial sites.

## 5. Conclusions

In this paper, a GCN-based LSTM autoencoder with a self-attention model for bearing fault diagnosis was proposed and evaluated using multivariate time series data. The proposed model was found to increase the accuracy of fault diagnosis by combining the GCN layer and the LSTM layer to extract important features from the frequency domain. In the data pre-processing step, data including various fault states and steady states were standardized, while features in the frequency domain were extracted through STFT conversion. Moreover, time series data were divided into fixed-length windows using the sliding window technique, which was used as input for the model.

The GCN-based LSTM autoencoder with self-attention model using multivariate time series data performed fault diagnosis by synthesizing various data obtained from multiple sensors. This model learned the spatial and structural relationship of the data using the GCN layer, and it simultaneously learned both long-term and short-term dependencies through the LSTM layer. The self-attention mechanism was added between the encoder and the decoder to highlight important features and synthesize various features to ultimately classify several types of bearings.

The comparative model performance evaluation indicated that the GCN-based LSTM autoencoder with self-attention model showed superior performance over other models in accuracy and F1 score in both the open CWRU dataset and the directly constructed fault simulator dataset. In particular, it was possible to detect abnormal conditions more accurately through reconstruction errors, which is an important advantage in quickly detecting and responding to various failures that occur in the manufacturing process. As a result of visually checking the classification performance of the model through the confusion matrix, the proposed model accurately classified most fault states and normal states, while there was a small number of incorrectly classified samples.

These findings can make an important contribution to early diagnosis and response to various failures that may occur in manufacturing sites, and they can be expected to substantially contribute to increasing the practicality of the model.

To further enhance the performance of the GCN-based LSTM autoencoder with the self-attention model proposed in this study, future research is planned in the following directions: Firstly, the development of a real-time fault diagnosis system based on the proposed model is planned. This is expected to improve the model’s real-time inference capabilities through real-time data collection, processing, and online learning. The real-time fault diagnosis system can quickly detect and respond to failures occurring in the manufacturing process, which can substantially contribute to improving productivity and reducing costs.

Next, the experiment in the current research was only conducted for specific bearing fault types, but in future research, we will conduct a study to increase the versatility of the model while including various failure types other than bearings. Through this, we will develop a general-purpose fault diagnosis model that can be used in various industrial environments as well as build a fault diagnosis system that can exhibit high performance even in various situations. We will also apply additional data augmentation techniques to increase the diversity of data. The data augmentation technique that is selected for use plays an important role in improving the generalizability of the model and enhancing the fault diagnosis performance in various situations. This will enable the model to learn various data patterns and facilitate more accurate fault diagnosis.

The practical implications of the proposed model include failure prevention and maintenance cost reduction, productivity improvement, reliable data-driven decision-making, industrial applicability, and implementation of smart manufacturing systems. By monitoring machine health in real time and diagnosing faults at an early stage, unexpected machine failures can be prevented, maintenance costs can be reduced, and overall productivity can be improved by minimizing production downtime. Reliable decisions can be made based on the data provided by the fault diagnosis system, which is very helpful for machine maintenance planning, resource allocation, production schedule adjustment, etc. Furthermore, the proposed model is not limited to a specific industry but can be applied in various manufacturing sectors, which can be utilized as a bearing fault diagnosis and preventive maintenance system in various industries such as automotive, aviation, energy, marine, etc. In this way, the proposed model can contribute to optimizing manufacturing processes and achieving innovative productivity by leveraging IoT, big data, and AI, which are key elements of Industry 4.0. These practical implications show that the proposed model can serve as an efficient and reliable fault diagnosis solution in various fields of manufacturing in the future.

Also, we plan to introduce a transfer learning technique to apply the model learned from one dataset to another. Through this, we intend to develop a model that can maintain high performance in various environments and conditions. Using this method will have the following advantages:

- Improved generalization: transfer learning allows the model to consistently perform highly on different datasets.
- Reducing training time: Applying already trained models to other datasets can reduce training time.
- Improved adaptability: allows the model to better adapt to new datasets or domains. This will be good for application to new manufacturing sites.

We plan to review practical applicability by applying the research results to actual industrial sites. Through such research, the performance of the model in the actual environment will be evaluated, and the model can be improved as necessary. This will increase the practicality of the model through verification in the actual industrial environment and contribute to solving problems in the actual field.

Finally, in addition to the GCN-based LSTM autoencoder with the self-attention model, we will conduct a fault diagnosis study using other recent deep learning models. We plan to compare the performance of various models and derive the optimal model. We expect that comparative studies with various deep learning models will make it possible to comprehensively evaluate the performance of the model and develop an optimal fault diagnosis model.

With these future research directions, we plan to further improve the performance of the models proposed in this study and eventually develop a practical fault diagnosis system that can be applied in various industrial environments. We believe that this will ultimately help increase the efficiency and reliability of the manufacturing industry, thereby contributing to productivity improvement and cost reductions.

## Figures and Tables

**Table 1:** System specifications.

Hardware Environment	Software Environment
CPU: Intel Core i7-8700K	Window 10, TensorFlow 2.11.0
GPU: NVIDIA GeForce RTX 3080	Python 3.7, CUDA 11.1

**Table 2:** CWRU dataset.

Fault Type (2HP)	Normal	Inner Fault			Ball Fault			Outer Fault		
**Diameter (inch)**	-	0.007	0.014	0.021	0.007	0.014	0.021	0.007	0.014	0.021
**Train Data**	34	34	34	34	34	34	34	34	34	34
**Validation Data**	11	11	11	11	11	11	11	11	11	11
**Test Data**	11	11	11	11	11	11	11	11	11	11

**Table 3:** Hyperparameters for the CWRU dataset.

Layers	Configurations	Input Shape	Output Shape
**Sliding Window & STFT**			
Sliding Window	Window_Size = 8, Step_Size = 4	(230, 9)	(56, 8, 9)
STFT	FS = 5000, Nperseg = 8	(56, 8, 9)	(56, 8, 5, 9)
**K-NN Layer**			
K-NN	K_Neighbors = 2	(56, 8, 5, 9)	(56, 8, 5, 9)
**GCN Layer**			
GCNConv_1	In_Channels = 9, Out_Channels = 16	(56, 8, 5, 9)	(56, 8, 5, 16)
GCNConv_2	In_Channels = 16, Out_Channels = 9	(56, 8, 5, 16)	(56, 8, 5, 9)
**Concatenate Data**			
Concatenate	-	(56, 8, 5, 9)	(56, 8, 45)
**LSTM autoencoder with Self-Attention**			
LSTM Encoder_1	Units = 64	(56, 8, 45)	(56, 8, 64)
LSTM Encoder_2	Units = 32	(56, 8, 64)	(56, 8, 32)
Attention Layer	-	(56, 8, 32)	(56, 32)
Repeat Vector	N = 8	(56, 32)	(56, 8, 32)
LSTM Decoder_1	Units = 32	(56, 8, 32)	(56, 8, 32)
LSTM Decoder_2	Units = 64	(56, 8, 32)	(56, 8, 64)
LSTM Decoder_3	Units = 128	(56, 8, 64)	(56, 8, 128)
TimeDistributed	Units = 10	(56, 8, 128)	(56, 8, 10)
Flatten Layer	-	(56, 8, 10)	(56, 80)
**Classifier**			
Dense_1	Units = 64, ReLU	(56, 80)	(56, 64)
Dropout_1	Rate = 0.5	(56, 64)	(56, 64)
Dense_2	Units = 32, ReLU	(56, 64)	(56, 32)
Dropout_2	Rate = 0.2	(56, 32)	(56, 32)
Output Dense	Units = 10, Softmax	(56, 32)	(56, 10)

**Table 4:** Fault simulator dataset.

Fault Type	Normal	Carriage	High-Speed	Lubrication	Corrosion
**Data Label**	0	1	2	3	4
**Train Data**	28,124	28,124	28,124	28,124	28,124
**Validation Data**	9374	9374	9374	9374	9374
**Test Data**	9375	9375	9375	9375	9375

**Table 5:** Hyperparameters for the fault simulator dataset.

Layers	Configurations	Input Shape	Output Shape
**Sliding Window & STFT**			
Sliding Window	Window_Size = 128, Step_Size = 64	(3,000,000, 4)	(46,875, 128, 4)
STFT	FS = 5000, Nperseg = 128	(46,875, 128, 4)	(46,875, 128, 65)
**K-NN Layer**			
K-NN	K_Neighbors = 2~8	(46,875, 128, 65, 4)	(46,875, 128, 65, 4)
**GCN Layer**			
GCNConv_1	In_Channels = 4, Out_Channels = 16	(46,875, 128, 65, 4)	(46,875, 128, 65, 16)
GCNConv_2	In_Channels = 16, Out_Channels = 4	(46,875, 128, 65, 16)	(46,875, 128, 65, 4)
**Concatenate Data**			
Concatenate	-	(46,875, 128, 65, 4)	(46,875, 128, 260)
**LSTM autoencoder with self-attention**			
LSTM Encoder_1	Units = 128	(46,875, 128, 260)	(46,875, 128, 128)
LSTM Encoder_2	Units = 64	(46,875, 128, 128)	(46,875, 128, 64)
LSTM Encoder_3	Units = 32	(46,875, 128, 64)	(46,875, 128, 32)
Attention Layer	-	(46,875, 128, 32)	(46,875, 32)
Repeat Vector	N = 128	(46,875, 32)	(46,875, 128, 32)
LSTM Decoder_1	Units = 32	(46,875, 128, 32)	(46,875, 128, 32)
LSTM Decoder_2	Units = 64	(46,875, 128, 32)	(46,875, 128, 64)
LSTM Decoder_3	Units = 128	(46,875, 128, 64)	(46,875, 128, 128)
TimeDistributed	Units = 5	(46,875, 128, 128)	(46,875, 128, 5)
Flatten	-	(46,875, 128, 5)	(46,875, 640)
**Classifier**			
Dense_1	Units = 64, LeakyReLU	(46,875, 640)	(46,875, 64)
Dropout_1	Rate = 0.5	(46,875, 64)	(46,875, 64)
Dense_2	Units = 32, LeakyReLU	(46875, 64)	(46,875, 32)
Dropout_2	Rate = 0.2	(46,875, 32)	(46,875, 32)
Output Dense	Units = 5, Softmax	(46,875, 32)	(46,875, 5)

**Table 6:** Model comparison using the CWRU dataset.

Model	Accuracy	Precision	Recall	F1 Score
LSTM	0.931	0.932	0.930	0.927
ConvLSTM	0.966	0.963	0.963	0.963
Autoencoder	0.900	0.905	0.900	0.899
LSTM Autoencoder	0.955	0.957	0.955	0.955
LSTM Autoencoder with Self-Attention	0.973	0.973	0.973	0.972

**Table 7:** Model comparison using the fault simulator dataset.

Model			Accuracy	F1 Score
LSTM	w/o STFT	w/o GCN	0.874	0.873
		with GCN	0.873	0.872
	with STFT	w/o GCN	0.868	0.867
		with GCN	0.981	0.980
ConvLSTM	w/o STFT	w/o GCN	0.893	0.891
		with GCN	0.838	0.837
	with STFT	w/o GCN	0.991	0.992
		with GCN	0.997	0.997
Autoencoder	w/o STFT	w/o GCN	0.494	0.494
		with GCN	0.804	0.804
	with STFT	w/o GCN	0.722	0.723
		with GCN	0.994	0.994
LSTM Autoencoder	w/o STFT	w/o GCN	0.437	0.414
		with GCN	0.793	0.792
	with STFT	w/o GCN	0.800	0.798
		with GCN	0.949	0.949
LSTM Autoencoder with Self-Attention	w/o STFT	w/o GCN	0.710	0.703
		with GCN	0.801	0.800
	with STFT	w/o GCN	0.830	0.820
		with GCN	0.999	0.999

## Data Availability

The data presented in this study are available in the following repositories: The CWRU dataset is openly available at the Case Western Reserve University Bearing Data Center website at https://engineering.case.edu/bearingdatacenter (accessed on 1 June 2024). The dataset extracted by the authors is available on GitHub at https://github.com/daeheetwo/Fault-Simulator-Data (accessed on 1 June 2024).
